# Gait Performance and Brain Activity Are Improved by Gait Automatization during Robot-Assisted Gait Training in Patients with Burns: A Prospective, Randomized, Single-Blinded Study

**DOI:** 10.3390/jcm13164838

**Published:** 2024-08-16

**Authors:** Seung Yeol Lee, Jisu Seo, Cheong Hoon Seo, Yoon Soo Cho, So Young Joo

**Affiliations:** 1 Department of Physical Medicine and Rehabilitation, College of Medicine, Soonchunhyang University Hospital, Bucheon 14584, Republic of Korea; shouletz@gmail.com; 2 Department of Rehabilitation Medicine, Hangang Sacred Heart Hospital, College of Medicine, Hallym University, Seoul 01000, Republic of Korea; jsseo219@gmail.com (J.S.); chseomd@gmail.com (C.H.S.); hamays@daum.net (Y.S.C.)

**Keywords:** lower extremity burns, robot-assisted gait training, functional near-infrared spectroscopy, automatization

## Abstract

**Background:** Patients with lower extremity burn injuries have decreased gait function. Gait dysfunctions are compensated by activation of executive areas such as the prefrontal cortex (PFC). Although robot-assisted gait training (RAGT) can improve gait function, the training mechanisms of RAGT are unknown. We aimed to determine the clinical effects of RAGT in patients with burns and investigate their underlying mechanisms. **Methods:** This single-blind, randomized controlled trial involved 54 patients with lower extremity burns. The RAGT group underwent RAGT using SUBAR^®^ and conventional training. The control (CON) group underwent only conventional training. The primary outcome was cortical activity measured using a functional near-infrared spectroscopy device before and after 8 weeks of training to confirm the compensatory effect of gait dysfunction. The secondary outcomes were the functional ambulation category (FAC) to evaluate gait performance, 6-min walking test (6 MWT) distance to measure gait speed, isometric force and range of motion (ROM) of lower extremities to evaluate physical function, and the visual analog scale (VAS) score to evaluate subjective pain during gait. **Results:** PFC activation during the gait phase in the RAGT group decreased significantly compared with that of the CON. The VAS score decreased and FAC score improved after 8 weeks of training in both groups. The 6 MWT scores, isometric strengths (the left knee flexor and bilateral ankle plantar flexors), and the ROMs (the extensions of bilateral hip and bilateral knee) of the RAGT group were significantly improved compared with those of the CON. RAGT improved gait speed, lower extremity ROMs, and lower extremity muscles strengths in patients with burns. **Conclusions:** The improvement in gait speed and cerebral blood flow evaluation results suggests that the automatization of gait is related to the treatment mechanism during RAGT.

## 1. Introduction

Gait is an automatic movement and an inherent skill. Decreased gait function is common in patients with lower extremity burns due to limited range of motion (ROM) or nerve injury, depending on the degree of burn [1]. In patients with nerve injury, a conscious effort is required to induce habitual behaviors such as gait; this phenomenon is known as reduced gait automaticity [2]. Patients with a decreased gait performance are at an increased risk of falls [3]. Therefore, gait training is essential to achieve good clinical outcomes in patients with burns. Repeating a normal gait pattern can enhance the function of neural circuits contributing to gait pacing [4]. Robot-assisted gait training (RAGT) is clinically useful for rehabilitating patients with central nervous system damage, such as stroke and spinal cord injury, and several studies have been conducted on the training mechanisms underlying improved gait function [5]. In patients with musculoskeletal diseases, positive effects on gait performance have been reported, attributed to reduced pain, inflammation, and atherogenic muscle inhibition through robot training [6,7]. Researches are being conducted on the application of robot training to improve gait function and gait speed in burn patients [8,9].

Currently, studies on the mechanisms by which RAGT improves gait performance in patients with central nervous system disorders are being conducted [10,11]. The gait is an automatic reaction. When automatic gait abnormalities occur due to nerve damage or muscle weakness, gait dysfunctions are compensated by activation of compensation circuits in executive areas such as the prefrontal cortex (PFC), premotor cortex, and supplementary motor areas [12]. Functional near-infrared spectroscopy (fNIRS) allows real-time brain activation analysis while the patient performs a dynamic task [13]. It offers the advantage of reduced sensitivity to motion artifacts, making it a promising tool for improving our understanding of brain activation during performance [14]. Therefore, fNIRS may enable the assessment of treatment effects and pathological mechanisms of pain or gait dysfunction [15,16].

This study aimed to confirm the clinical effects and the therapeutic mechanism of RAGT that induces improvement in gait function, which has not been performed so far, through evaluation of physical performance and brain network after RAGT and conventional gait training in patients with burn injury. 

## 2. Methods

### 2.1. Patient Selection and Ethical Considerations

This was a single-blind, prospective, randomized controlled trial. This study was registered at ClinicalTrials.gov (accessed on 3 August 2023, identifier: NCT05988905). The study was conducted in accordance with the Declaration of Helsinki, and approved by the Ethics Committee of Hangang Sacred Heart Hospital (HG 2019-004). Written informed consent was obtained from all participants. Patients who underwent skin grafting at our institution between January 2019 and December 2021 were included. 

**Figure 1:**
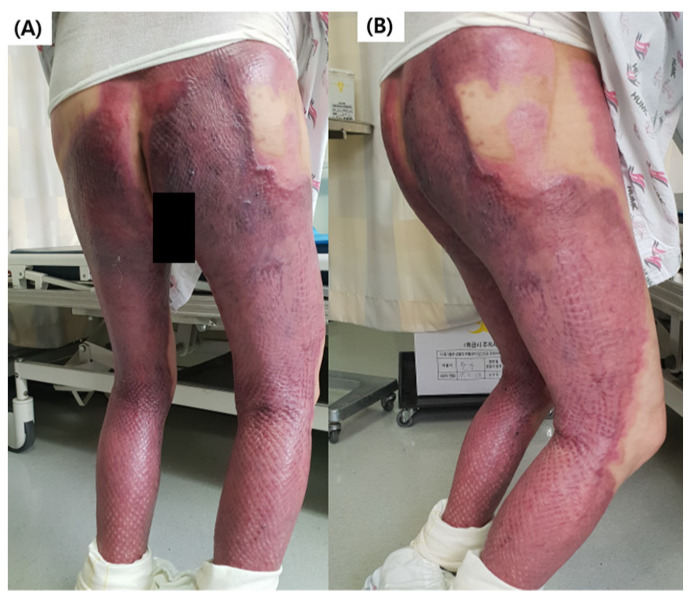
A participant of this study. (**A**) Posterior and (**B**) Lateral views.

The specific inclusion criteria were as follows: with deep second or full thickness burns of >50% of the body surface area of both lower extremities (Figure 1), >18 years, with a ≤1 functional ambulation category (FAC) score of ≤3, and lower limb lengths that can be worn with the robot (calf length from 440 mm to 510 mm, thigh length from 390 mm to 460 mm). The exclusion criteria were as follows: patients with cognitive disorders, intellectual impairment, serious cardiac dysfunction, problems with weight bearing due to unstable fractures, body weight ≥ 100 kg, which is the level at which you can ride on the robot and train, severe fixed contracture, skin disorders that RAGT and conventional training could worsen, and patients with severe pain unable to undergo rehabilitation programs. Numbers were assigned according to the order of admissions of 60 burn patients who satisfied all of the aforementioned criteria. A computer program was then used to divide the patients into the RAGT group (*n* = 30) and control (CON) group (*n* = 30). 

### 2.2. Interventions

Both groups received individually tailored gait training based gait function, lower extremity joint ROM, and muscle strength at a rehabilitation center for hospitalized patients. Both training programs consisted of 40 sessions for 8 weeks (5 days/week). Both groups received gait-related training for 60 min per day at the same duration and frequency.

#### 2.2.1. CON Group Training

Conventional training comprising active ROM exercises, weight-bearing training, and manual lymphatic drainage, and hypertrophic scar care was performed daily throughout the rehabilitation period. The CON group patients received 30 min of conventional training each in the morning and afternoon. 

#### 2.2.2. RAGT Group Training

**Figure 2:**
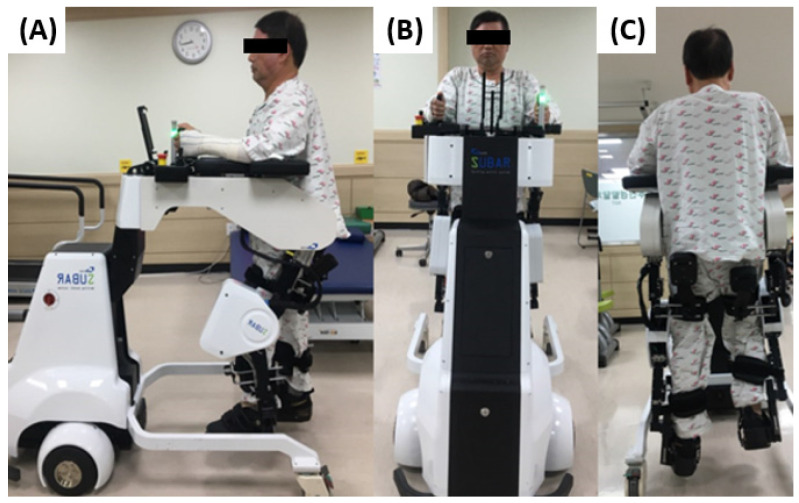
Robot-assisted gait training of a patient with burn injuries. (**A**) Lateral, (**B**) anterior, and (**C**) posterior views.

SUBAR^®^ (Cretem, Anyang-si, Republic of Korea) is an exoskeleton robot with a footplate that assists with gait movement. The length of the thigh and lower leg of the patient was measured, and the exoskeleton length of SUBAR^®^ was adjusted to suit the patient (Figure 2). A therapist, who is experienced with SUBAR^®^, adjusted the angle of the knee joint, step length, and gait speed during training. The three parameters (knee joint angle, step length, and gait speed) were adjusted to ensure comfortable training for the patients according to the results of gait speed and joint ROM evaluation [8,9]. Each session comprised a 30-min robot training period, with additional setup and post-training evaluation time. The RAGT group patients underwent 30 min of conventional training along with robot training.

### 2.3. Measuring the Cortical Activity

**Figure 3:**
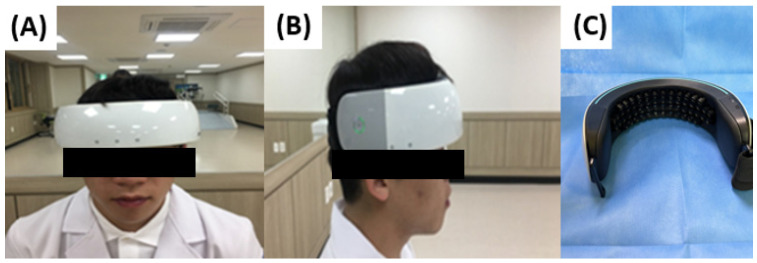
(**A**) Anterior view of the functional near-infrared spectroscopy (fNIRS), (**B**) lateral view, and (**C**) fNIRS laser and detector.

The outcome evaluations were performed before and immediately after 8 weeks of training. Outcome measurements and data analysis were performed by a trained and blinded outcome assessor who was not involved in the training. The primary outcome was PFC activity in both groups. The evaluator measured the gait-related cortical activity of the patients using an fNIRS device (NIRSIT^®^; OBELAB Inc., Seoul, Korea) during the resting and gait phases. The fNIRS device was fastened to the head using elastic straps inside a plastic cap. The middle of the marker was aligned with the middle of the eyes (nasion in the 10–20 system), and the bottom line of the device was positioned just above the participants’ eyebrows [15,16]. This system uses 24 laser sources (780/850 nm; maximum power under 1 mW) and 32 photodetectors to measure the signals from the PFC area. The device has 48 channels with a 3-cm distance between the laser and detector (Figure 3). Each cycle comprised three phases of 60 s each (stabilization, rest, and gait), and the participants were instructed to repeat five cycles. Gait is defined as walking at a comfortable pace. Five trial results for each participant were averaged for each condition. After the resting period, the “start” instruction was given. The participants walked back and forth on a 30-m walkway for 60 s. The rest period before the gait period served as the baseline reference for oxyhemoglobin (HbO_2_) PFC perfusion. Cortical activity was measured by evaluating the relative changes in HbO_2_ and deoxyhemoglobin (HbR) levels. Changes in HbO_2_ and HbR concentrations, which are driven primarily by synaptic activity, were estimated with the modified Beer-Lambert [13]. The NIRST Analysis Tool v2.1 was used to analyze the fNIRS data in MATLAB.

### 2.4. Assessing Treatment Effects and Functional Recovery

A visual analog scale (VAS) was used to rate the degree of subjective pain during gait movement to assess the treatment effect: 0 points were assigned when no pain was noted, and 10 points were assigned when unbearable pain was noted. Functional ambulatory category (FAC) score and 6-min walking test (6 MWT) distance were measured to evaluate functional recovery. The FAC was evaluated using six scales. A score of zero indicated that the patient could not walk or could only walk with the assistance of two people. A score of five indicated that the patient could walk independently. The 6 MWT followed standardized guidelines, and the walking course was 20 m. Patients were instructed to walk as far as possible in 6 min [17]. Outcomes were measured by a physical therapist before and after 8 weeks of training. Isometric muscle strengths of the hip extensors, hip flexors, knee extensors, knee flexors, ankle dorsiflexors, and ankle plantar flexors of bilateral lower extremities were measured using microFET II™ (Hoggan Health Industries, Draper, UT, USA) [18]. Each muscle strength was evaluated twice, and each evaluation was performed for 3–5 s, with an interval of 30 s between measurements. A higher value was selected in the evaluation. The active ROMs of bilateral lower extremities were measured using a Jamar stainless-steel goniometer (Sammons Preston, Rolyan, Bolingbrook, IL, USA) following a standardized technique [19].

### 2.5. Statistical Analyses

All statistical analyses were performed using SPSS version 23 (IBM Corp., Armonk, NY, USA). Values are presented as the mean ± standard deviation. The assessor tested the differences between the RAGT group and CON group at baseline using Fisher’s exact test for sex and burn type, and the Mann–Whitney test for age, total body surface area (TBSA), and the duration between injury and initiation of training. A comparison of pretreatment scores and post-treatment scores of each group was performed using the Wilcoxon signed-rank sum test. Intergroup comparisons were made using the Mann–Whitney test at 8 weeks after the normality test, with a significance level of *p* < 0.05. Accordingly, eighteen patients were required in each group to provide 80% power for efficacy evaluation, setting the α level at 0.05. We calculated that a minimum 52 patients were needed, considering a 30% dropout rate.

## 3. Results

### 3.1. Patient Demographics

**Figure 4:**
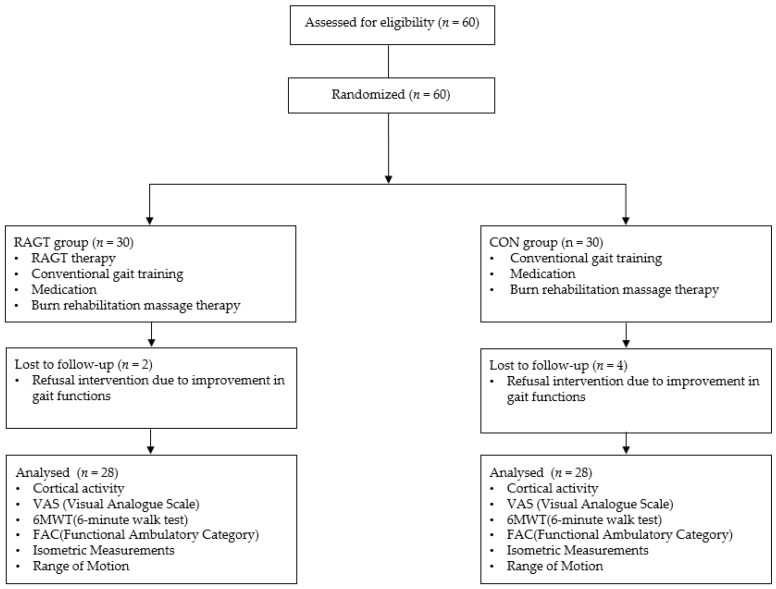
Consolidated Standard of Reporting Trials flow diagram.

Two patients in the RAGT group and four patients in the CON group dropped out of the study because their gait function improved and they refused gait training or did not undergo serial assessment. The remaining patients were randomly divided into two groups (28 patients in the RAGT group and 26 patients in the CON group) (Figure 4). A total of 54 patients who underwent 40 RAGT or conventional training sessions were evaluated in this study. The mean age of the patients was 51.21 ± 11.45 years and 51.85 ± 12.34 years in the RAGT and CON groups, respectively. The demographic characteristics of the patients are presented in Table 1.

### 3.2. Outcomes of PFC Activation

**Figure 5:**
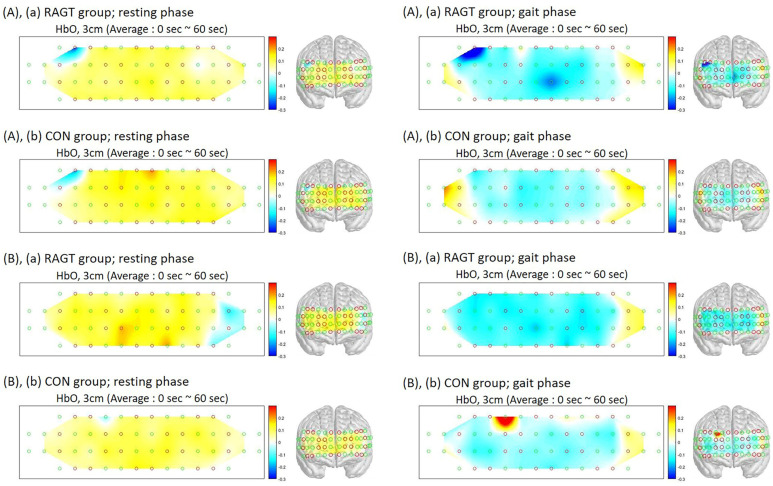
Reconstructed DOP images of oxyhemoglobin (HbO_2_). (**A**) (a) Robot-assisted gait training (RAGT) group and (b) CON group before training, (**B**) (a) RAGT group and (b) CON group after 8 weeks of training.

The PFC HbO_2_ analysis showed that the measurements of PFC activation during the gait phase in the RAGT group decreased significantly compared to those in the CON group (*p* = 0.002) (Table 2 and Figure 5). Analysis of the HbR in the PFC showed no significant difference between the RAGT and CON groups before and after training.

### 3.3. Outcomes of Physical Functions

Patients in both groups underwent 8 weeks of training. The VAS score significantly decreased from 8.43 ± 0.92 before training to 5.50 ± 1.62 points (*p* < 0.01) after training, and from 8.54 ± 0.86 to 5.69 ± 1.16 points (*p* < 0.01) in the RAGT and CON groups, respectively (Table 3). The FAC score increased from 0.79 ± 0.42 before training to 3.29 ± 0.71 points (*p* < 0.01) after training, and from 0.62 ± 0.50 to 3.08 ± 0.74 points (*p* < 0.01) in the RAGT and CON groups, respectively. In both groups, improvement was observed after training in the VAS and FAC scores, but the difference was not significant (Table 3). The 6 MWT score increased from 283.57 ± 112.98 m before training to 377.86 ± 80.39 m (*p* < 0.01) after training, and from 265.38 m ± 119.37 to 323.85 ± 92.57 m (*p* = 0.13) in the RAGT and CON groups, respectively. No statistically significant differences were observed in VAS scores (5.50 ± 1.62 and 5.69 ± 1.16) and FAC scores (3.29 ± 0.71 and 3.08 ± 0.74) after 8 weeks of training in the RAGT and CON group. After 8 weeks of training, the 6 MWT score (377.86 ± 80.39 m) of the RAGT group were statistically increased compared with that of the CON group (323.85 ± 92.57 m) (*p* = 0.02) (Table 3).

The isometric strengths of the left knee flexors (*p* = 0.03), right ankle plantar flexors (*p* = 0.01), and left ankle plantar flexors (*p* = 0.01) after training were significantly improved compared to those before training in the RAGT group (Table 3). The ROMs of the right hip extensions (*p* = 0.04), right knee extensions (*p* = 0.01), and left knee extensions (*p* = 0.03) were significantly improved compared with those before training in the RAGT group. There were no significant improvements in the isometric strengths and ROMs of the bilateral hip, knee, and ankle muscles after training in the CON group (Table 3). Comparing the isometric strengths after training revealed a significant increase in the left knee flexors (*p* = 0.04), right ankle plantar flexors (*p* = 0.03), and left ankle plantar flexors (*p* = 0.01) in the RAGT group (Table 3). Comparing the ROMs after training showed a significant increase in the right hip extensions (*p* = 0.01), left hip extensions (*p* = 0.03), right knee extensions (*p* = 0.01), and left knee extensions (*p* = 0.01) in the RAGT group (Table 3).

None of the patient experienced adverse events, such as skin abrasions and worsening of joint pain, during training. No surgery-related adverse events were recorded.

## 4. Discussion

In the present study, improvements in subjective pain during gait and gait performance were observed in both groups. In RAGT group, it was confirmed that there were statistically significant improvement in lower joint ROMs (hip extension and knee extension), lower extremity muscle strengths (knee flexor and ankle plantar flexor), and gait speed compared to the CON group. The improvement in gait speed and cerebral flow evaluation results suggests that the automatization of gait is related to the treatment mechanism during RAGT. 

The primary motor area, cerebellum, and spinal cord control automatic gait. This pathway is activated in less challenging gait situations. When the automatic gait is not feasible, during more challenging tasks or decreased balance or weakened muscle power, executive control involving the PFC, premotor cortex, and supplementary motor cortex is activated [20]. Executive gait control can be estimated by recording PFC activation using fNIRS [13]. The additional effort required for a fast-paced gait allows differences in functionality to emerge in patients with reduced automatization [21]. In patients with reduced gait function, the demand for attention increases during above-ground gait, resulting in PFC activation. PFC overactivation in less functional patients may be due to loss of stepping automaticity [22]. However, an increase in attention compensates for gait automaticity [23]. A higher PFC activity during gait confirms a compensatory mechanism for functional decline and reduced gait automaticity [24]. More intense and extensive activation of the cortical motor area occurs when training used the active mode, which is required patient effort, rather than the 100% robot-dependent passive mode [6]. During training to improve gait function, stimulation initially improves gait function by increasing the PFC activity. Repeated training induces automatic gait control as an adaptation and does not require PFC activation [12]. A previous study used fNIRS after robotic training to observe the improvement in gait function with increased brain activation of the affected motor areas compared to conventional training in patients with stroke [25]. Significant decrease in compensatory activation of the PFC observed was observed after RAGT training in this study.

Improvements in subjective pain during gait and gait performance were observed in both groups. In RAGT group, it was confirmed that there were statistically significant improvement in lower joint ROMs, lower extremity muscle strengths, and gait speed. Gait training enables patients to walk repetitively with a normal gait pattern [26]. Robot training improves gait performance and speed, lower limb strength, and joint ROM of the lower extremities in patients [8,9]. Several studies have been conducted on the clinical usefulness of RAGT [27,28,29,30]. In addition to the clinical effects, studies on the mechanism of improved gait function are also being actively conducted. Repetitive, task-specific robot-assisted training improves motor impairment by motor learning in neurological motor rehabilitation [31,32,33]; this approach of intensively engaging the impaired limb promotes neuroplasticity and the carryover of motor improvements [34,35]. RAGT also offers secondary benefits, including improved postural control by stimulation of the trunk muscles [36,37]. In addition, intense somatosensory stimulation can aid in the recovery of gait automatization and speed [2]. Gait performance refers to a motor-conditioned reflex guided by muscle sense differentiation, consolidation and stabilization, and automatization [38]. Gait performance is also improved when robot training is performed at various intensities, such as climbing stairs or walking on a slope [39,40]. This therapeutic effect is due to stimulation of the normal gait physiological cycle through sensory stimulations [38]. Innovative mechanical and motorized devices can provide intensive, repetitive, task-specific, and multisensory training, thereby driving neuroplastic changes and enhancing motor outcomes. 

The present study had some limitations. The training was only performed for 8 weeks, and patients were followed up only during the early rehabilitation period. Moreover, only 54 patients were evaluated in this study. Therefore, further studies evaluating functional outcomes in a larger numbers of patients are required to confirm the efficacy of RAGT. This study only observed PFC activity; therefore, research should be conducted to evaluate gait pathways, including imaging techniques such as Magnetic Resonance Imaging that can help observe subcortical areas. To evaluate gait automatization, investigators must use gait analysis to study the measurement of temporospatial gait parameters, including stride length and stance time. 

## 5. Conclusions

In conclusion, this study demonstrated that robot training induces positive clinical effects in patients with lower extremity burns, and gait automatization may be involved in the training mechanisms of RAGT. To the best of our knowledge, this is the first study to observe the treatment mechanism through which robotic training improves gait function in patients with burns.

## Figures and Tables

**Table 1:** Demographic data of the patients.

	RAGT Group (*n* = 28)	CON Group (*n* = 26)	*p*-Value
Male: female	24:4	22:4	0.91
Age (years)	51.21 ± 11.45	51.85 ± 12.34	0.84
TBSA (%)	32.57 ± 19.36	30.38 ± 15.67	0.76
Mechanism of burn, n FB: SB: CB: EB	18:4:4:2	14:6:2:4	0.48
Duration (days) between burn injury and therapy	93.50 ± 37.83	81.77 ± 28.86	0.18
VAS	8.43 ± 0.92	8.54 ± 0.86	0.59
FAC	0.79 ± 0.42	0.62 ± 0.50	0.18
6 MWT (m)	283.57 ± 112.98	265.38 ± 119.37	0.73
Isometric measurements (Nm)			
Hip flexor, right	25.29 ± 7.17	26.69 ± 4.71	0.31
Hip flexor, left	23.86 ± 7.14	25.38 ± 5.61	0.47
Hip extensor, right	18.64 ± 7.79	17.46 ± 7.08	0.47
Hip extensor, left	17.62 ± 8.37	15.62 ± 8.18	0.33
Knee flexor, right	17.36 ± 7.40	18.85 ± 4.66	0.16
Knee flexor, left	17.43 ± 6.86	18.62 ± 7.45	0.51
Knee extensor, right	22.17 ± 9.64	24.77 ± 6.07	0.16
Knee extensor, left	22.64 ± 7.35	23.38 ± 7.46	0.47
Ankle dorsiflexor, right	17.21 ± 8.80	18.23 ± 6.03	0.51
Ankle dorsiflexor, left	16.41 ± 8.89	16.31 ± 8.18	0.90
Ankle plantarflexor, right	16.00 ± 6.99	15.77 ± 4.67	0.78
Ankle plantarflexor, left	15.01 ± 7.87	15.23 ± 6.39	0.92
Range of Motion (degree)			
Hip flexion, right	94.57 ± 15.69	99.62 ± 1.36	0.12
Hip flexion, left	95.71 ± 15.74	98.85 ±11.34	0.47
Hip extension, right	19.93 ± 7.06	22.62 ± 4.59	0.87
Hip extension, left	21.57 ± 5.68	22.23 ± 5.63	0.70
Knee flexion, right	122.29 ± 19.62	123.85 ± 17.37	0.33
Knee flexion, left	117.29 ± 27.10	124.31 ± 21.14	0.44
Knee extension, right	−3.43 ± 7.05	−1.15 ± 3.29	0.09
Knee extension, left	−5.14 ± 13.51	−4.62 ± 7.61	0.73
Ankle dorsiflexion, right	16.07 ± 5.39	16.92 ± 5.48	0.45
Ankle dorsiflexion, left	16.36 ± 5.88	14.85 ± 6.36	0.49
Ankle plantarflexion, right	37.50 ± 4.91	38.46 ± 3.77	0.47
Ankle plantarflexion, left	37.14 ± 6.30	34.92 ± 8.35	0.37

RAGT, robot-assisted gait training; CON: conventional training; TBSA: total body surface area; FB: flame burn; EB: electrical burn; SB, scaling burn; CB: contact burn; VAS: visual analog scale; FAC: functional ambulatory category; 6 MWT, 6-min walking test.

**Table 2:** Comparison of HbO_2_ and HbR levels between the RAGT and CON groups.

	Before Training			After 8 Weeks of Training		
	RAGT Group	CON Group	*p*-Value	RAGT Group	CON Group	*p*-Value
Resting phase						
HbO_2_	0.00150 ± 0.00454	0.00046 ±0.00047	0.95	0.00046 ± 0.00062	0.00024 ± 0.00061	0.30
HbR	−0.00032 ± 0.00094	0.00000 ± 0.00015	0.17	−0.00011 ± 0.00016	0.00001 ± 0.00039	0.19
Gait phase						
HbO_2_	−0.00060 ± 0.00076	−0.00032 ± 0.00121	0.10	−0.00079 ± 0.00062	−0.00013 ± 0.00074	0.002 *
HbR	0.00001 ± 0.00036	0.00020 ± 0.00048	0.21	0.00013 ± 0.00042	0.00005 ± 0.00030	0.89

HbO_2_, oxyhemoglobin; HbR, deoxyhemoglobin; RAGT, robot-assisted gait training; CON, conventional training. Values are presented as mean ± standard deviation. The *p*-values for between-condition differences were calculated using the Wilcoxon signed-rank sum test (* *p* < 0.05).

**Table 3:** Comparison of scores obtained before and after training.

	RAGT Group (*n* = 28)			CON Group (*n* = 26)			
	Before Training	After Training	*p*-Value	Before Training	After Training	*p*-Value	Intergroup *p*-Value after Training
VAS	8.43 ± 0.92	5.50 ± 1.62	<0.01 *	8.54 ± 0.86	5.69 ± 1.16	<0.01 *	0.26
FAC	0.79 ± 0.42	3.29 ± 0.71	<0.01 *	0.62 ± 0.50	3.08 ± 0.74	<0.01 *	0.30
6 MWT	283.57 ± 112.98	377.86 ± 80.39	<0.01 *	265.38 ± 119.37	323.85 ± 92.57	0.13	0.02 **
Isometric measurements (Nm)							
Hip flexor, right	25.29 ± 7.17	25.32 ± 10.09	0.99	26.69 ± 4.71	21.65 ± 5.69	<0.01 *	0.09
Hip flexor, left	23.86 ± 7.14	23.00 ± 10.67	0.68	25.38 ± 5.61	23.42 ± 7.04	0.30	0.34
Hip Extensor, right	18.64 ± 7.79	20.11 ± 8.91	0.51	17.46 ± 7.08	17.12 ± 4.11	0.84	0.12
Hip extensor, left	17.62 ± 8.37	16.29 ± 7.24	0.98	15.62 ± 8.18	19.46 ± 6.91	0.13	0.12
Knee flexor, right	17.36 ± 7.40	22.00 ± 7.28	0.05	18.85 ± 4.66	18.92 ± 8.20	0.60	0.06
Knee flexor, left	17.43 ± 6.86	23.14 ± 8.49	0.03 *	18.62 ± 7.45	18.46 ± 7.06	0.73	0.04 *
Knee extensor, right	22.17 ± 9.64	25.32 ± 6.80	0.16	24.77 ± 6.07	23.73 ± 10.53	0.35	0.27
Knee extensor, left	22.64 ± 7.35	24.75 ± 6.02	0.15	23.38 ± 7.46	22.58 ± 8.55	0.19	0.23
Ankle dorsiflexor, right	17.21 ± 8.80	21.21 ± 7.24	0.08	18.23 ± 6.03	17.23 ± 9.76	0.49	0.06
Ankle dorsiflexor, left	16.41 ± 8.89	20.11 ± 8.90	0.12	16.31 ± 8.18	16.19 ± 10.00	0.63	0.13
Ankle plantarflexor, right	16.00 ± 6.99	21.50 ± 9.09	0.01 *	15.77 ± 4.67	16.46 ± 7.13	0.77	0.03 **
Ankle plantarflexor, left	15.01 ± 7.87	21.71 ± 8.78	0.01 *	15.23 ± 6.39	15.69 ± 7.15	0.93	0.01 **
Range of motion (degree)							
Hip flexion, right	94.57 ± 15.69	93.93 ± 19.64	0.96	99.62 ± 1.36	99.00 ± 2.77	0.28	0.96
Hip flexion, left	95.71 ± 15.74	94.43 ± 19.52	0.67	98.85 ±11.34	96.31 ± 6.94	0.33	0.28
Hip extension, right	19.93 ± 7.06	23.54 ± 5.67	0.04 *	22.62 ± 4.59	18.81 ± 6.57	0.02 *	0.01 **
Hip extension, left	21.57 ± 5.68	24.46 ± 5.44	0.11	22.23 ± 5.63	19.58 ± 6.11	0.10	0.03 **
Knee flexion, right	122.29 ± 19.62	128.32 ± 16.75	0.18	123.85 ± 17.37	121.96 ± 25.02	0.77	0.50
Knee flexion, left	117.29 ± 27.10	130.43 ± 20.56	0.10	124.31 ± 21.14	116.85 ± 29.13	0.35	0.06
Knee extension, right	−3.43 ± 7.05	1.93 ± 7.97	0.01 *	−1.15 ± 3.29	−2.08 ± 4.81	0.29	0.01 **
Knee extension, left	−5.14 ± 13.51	1.93 ± 7.97	0.03 *	−4.62 ± 7.61	−2.38 ± 4.81	0.17	0.01 **
Ankle dorsiflexion, right	16.07 ± 5.39	23.43 ± 28.06	0.35	16.92 ± 5.48	14.00 ± 8.20	0.12	0.30
Ankle dorsiflexion, left	16.36 ± 5.88	22.18 ± 28.52	0.99	14.85 ± 6.36	13.96 ± 8.06	0.38	0.51
Ankle plantarflexion, right	37.50 ± 4.91	36.71 ± 10.49	0.77	38.46 ± 3.77	36.15 ± 6.46	0.11	0.15
Ankle plantarflexion, left	37.14 ± 6.30	35.14 ± 11.20	0.48	34.92 ± 8.35	36.77 ± 5.85	0.33	0.69

TBSA, total body surface area; VAS: visual analog scale; FAC: functional ambulatory category; 6 MWT, 6-min walking test; * *p* < 0.05, Wilcoxon signed-rank sum test; ** *p* < 0.05, Mann–Whitney U test.

## Data Availability

The datasets used and/or analysed during the current study are available from the corresponding author on reasonable request.
